# Reassessing type 2 diabetes in adolescents and its management strategies based on insulin resistance

**DOI:** 10.3389/fendo.2024.1377918

**Published:** 2024-06-19

**Authors:** QianYou Jia, YanMin Zhang, BaoFeng Zhang, XueDong An

**Affiliations:** ^1^ Department of Pediatrics, Rizhao Hospital of Traditional Chinese Medicine, Rizhao, China; ^2^ Department of Endocrinology & Diabetes Vascular Function Laboratory, Guang’anmen Hospital of China Academy of Chinese Medical Sciences, Beijing, China

**Keywords:** youth-onset diabetes mellitus, insulin resistance, obesity, mechanisms, management strategies, diabetes remission

## Abstract

With changes in lifestyle behaviors, including dietary structure and habits, the prevalence of Youth-onset Type 2 Diabetes Mellitus (YODM) has increased 2 to 3 times compared to 30 years ago. YODM patients experience complications earlier, progress faster, and exhibit more severe symptoms. However, limited and inconclusive direct evidence, coupled with poor patient compliance, poses challenges in the clinical management of YODM. Apart from the continuous decline in pancreatic β-cell function and quantity, tissue-specific insulin resistance (IR) is also a typical characteristic of YODM. The main mechanisms of IR in YODM involve different aspects such as obesity, dietary imbalance, abnormal substance metabolism, chronic inflammation, oxidative stress, and hormonal fluctuations during adolescence. For the comprehensive management of YODM, besides achieving good control of blood glucose levels, it may be necessary to apply the most appropriate methods considering the uniqueness of the patient population and the specifics of the disease. Early identification and detection of the disease are crucial. Precise screening of patients with well-functioning pancreatic insulin β-cells, primarily characterized by IR and obesity, represents the population most likely to achieve diabetes remission or reversal through lifestyle modifications, medications, or even surgical interventions. Additionally, considering potential emotional disorders or the impact of adolescent hormones in these patients, health education for patients and caregivers is essential to make them aware of the long-term benefits of well-controlled blood glucose. In conclusion, adopting comprehensive management measures to achieve diabetes remission or reversal is the ideal goal. Controlling high blood glucose, obesity, and other risk factors related to diabetes complications is the next priority to delay the occurrence and progression of complications. A comprehensive perspective on IR provides insights and references for identifying YODM and its management strategies.

## 1 Introduction

The International Diabetes Federation pointed out in its 2021 conference that globally, 537 million adults (20–79 years old) are affected by Diabetes Mellitus (DM), and it is projected to rise to 784 million by the year 2045. Changes in dietary structure (consumption of high-calorie foods) and lifestyle habits (such as staying up late and insufficient physical activity) contribute to a trend of younger onset Type 2 Diabetes Mellitus (T2DM). Research indicates that the prevalence of Youth-onset Type 2 Diabetes Mellitus (YODM), that occurs during adolescence (typically between the ages of 10 and 18), has increased 2 to 3 times compared to 30 years ago, with an estimated 41,600 new cases worldwide in 2021, particularly higher rates reported in China, India, and the United States (1–3).

While comprehensive management for adult DM patients is becoming increasingly standardized across various aspects such as diagnosis, monitoring, prevention, and treatment, the same cannot be said for YODM. This is primarily due to its unique characteristics, including the need for improved systemic management measures, rapid disease progression, poor compliance, and prominent psychological and emotional challenges. Data from “Healthy People 2020” reveals that only 27.1% of adolescents meet the recommended levels of physical activity, and just 31.7% get adequate sleep (4). Dietary imbalances are common among YODM, impacting obesity and psychosocial functionality. The Treatment Options for Type 2 Diabetes in Adolescents and Youth Study (TODAY) indicates that 20% and 6% of young individuals exhibit subclinical or clinical binge-eating behavior (5). These unfavorable lifestyle habits contribute to the rising incidence of YODM, and information about its pathophysiology is mostly derived from adult studies (6). Considering the unique nature of the affected population, YODM patients face a higher risk of depression and other mental health disorders, with an estimated depression prevalence exceeding 20% (7, 8). This not only affects blood glucose control but also directly contributes to lower patient compliance (9). In comparison to adult T2DM, YODM has an earlier onset, a faster decline in pancreatic β-cell function, and a higher likelihood of complications if not controlled promptly (10). Approximately 8 years after YODM diagnosis, 72% of T2DM patients experience at least one complication (11). As a result, YODM has become an increasingly serious global health issue for adolescents and young people, demanding more attention (12–14).

In addition to the continuous decline in pancreatic β-cell function and quantity, tissue-specific insulin resistance (IR) is also a typical characteristic of T2DM, including YODM, holding a significant position in its occurrence and development (15–17). Therefore, focusing on IR allows for the transfer of some T2DM management strategies to YODM. Simultaneously, expectations for the treatment goals of some YODM patients should be elevated, including diabetes remission, defined as having an HbA1c below 6.5% for at least one year without the use of glucose-lowering medications according to the standards recommended by the American Diabetes Association (ADA) and the European Association for the Study of Diabetes (EASD). And the most ideal state, crucial for long-term improvements in quality of life and survival (18). This is followed by controlling risk factors to delay the occurrence and progression of complications. However, the immediate priority lies in the early identification of adolescent IR or even YODM, enabling the prompt alteration of metabolic abnormalities. Thus, early detection and intensive management of YODM are paramount (19). We first summarize the clinical characteristics (specificities, risk factors) of YODM, focusing on IR, and then explores management strategies for YODM, aiming to provide reference points for the clinical understanding and management of YODM.

## 2 Comprehensive and accurate understanding of YODM is key to developing more effective comprehensive management strategies

### 2.1 More specific characteristics of YODM compared to adult T2DM

Both adult T2DM and YODM share common risk factors, pathological changes, and clinical manifestations as they both belong to the diabetes category. Common risk factors include obesity, family history, shared pathological changes such as reduced insulin sensitivity and insufficient insulin secretion, and common clinical manifestations like polydipsia, polyuria, increased appetite, and fatigue (20). However, due to the younger age of the YODM patient population, they exhibit unique clinical features, making management more challenging, disease progression faster, and the condition more severe (21).

#### 2.1.1 Earlier onset of complications, faster disease progression, and more severe symptoms in YODM patients

Long-term follow-up reports from the TODAY study (average diabetes duration of 13.3 ± 1.8 years) show that YODM patients have higher rates of diabetic nephropathy (54.8%), neuropathy (32.4%), and retinopathy (13.7%) (22). The SEARCH study also indicates that compared to Type 1 Diabetes Mellitus (T1DM) patients, YODM patients have higher occurrence rates of diabetic nephropathy (19.9% vs. 5.8%), retinopathy (9.1% vs. 5.6%), and peripheral neuropathy (17.7% vs. 8.5%) (11). Although diabetic ketoacidosis is not a common initial presentation in YODM, its frequency can be as high as 3% to 11%, with high blood glucose and hyperosmolarity states being rare (23, 24).

#### 2.1.2 Limited and challenging clinical management due to insufficient evidence and poor compliance in YODM patients

There are relatively few interventional clinical studies focused on YODM, with major studies including the TODAY study and SEARCH study. Ethical considerations, along with the transitional phases YODM patients undergo in physiological, psychological, and social aspects, make it challenging to conduct diagnostic and interventional clinical studies due to the significant emotional disturbances exhibited by patients. Despite partial references to the pathophysiology evidence from adult diabetes studies (6), the scarcity of such studies poses limitations. A study in Korea assessing the prevalence and characteristics of YODM using the National Health Insurance Service (NHIS) database highlighted non-adherence to treatment and low compliance as major obstacles in managing YODM patients (19). Adolescents (10 to 20 years old) are more likely to display medical non-adherence, and data indicates that the younger the age, the lower the blood glucose control rate in T2DM patients (25–27). YODM patients relying solely on lifestyle modifications and metformin for blood glucose control exhibit a high rate of poor blood glucose control, reaching 51.7% (28). Therefore, effective strategies and interventions are needed to manage YODM and reduce the risks of microvascular and macrovascular complications, addressing compliance issues more effectively than seen in adult patients.

### 2.2 Risk factors for the occurrence and development of YODM

Critical driving factors for the prevalence of YODM include both genetic factors inherited from parents and acquired risk factors related to oneself, such as obesity, unhealthy lifestyle habits, and psychological factors.

#### 2.2.1 Genetic factors inherited from parents

For the majority of YODM cases (92%), genetic factors should be considered, particularly for patients with atypical presentations and/or a positive family history (23, 29). If one parent has T2DM, the estimated lifelong risk for their child developing T2DM is 40%, and if both parents have T2DM, the risk increases to as much as 70% (30, 31). Research also suggests that whether maternal diabetes is diagnosed during pregnancy or post-pregnancy, it is associated with poor blood glucose control and reduced glomerular filtration rate in YODM patients (32). Interestingly, a study in Denmark involving 2,448,753 individuals found that offspring of mothers with gestational hypertension (HR=1.37) or preeclampsia (HR=1.62) have a higher risk of developing T2DM (33), possibly due to intrauterine growth restriction caused by imbalanced nutrient intake leading to fetal adipose tissue and pancreatic β-cell dysfunction (34). The Progress in Genetic studies of youth-onset diabetes (ProDiGY) consortium conducted a genome-wide association study on YODM, identifying seven crucial loci in the genome, including rs7903146 in TCF7L2, rs72982988 near MC4R, rs200893788 in CDC123, rs2237892 in KCNQ1, rs937589119 in IGF2BP2, rs113748381 in SLC16A11, and rs2604566 in CPEB2, which may play a significant role in early detection of YODM in the future (35).

#### 2.2.2 Acquired risk factors

The most crucial acquired risk factor for the development of YODM is obesity, primarily attributed to unhealthy dietary or exercise habits (36, 37). In recent years, with the widespread adoption of fast food culture and the increased consumption of ultra-processed foods, the proportion of adolescents consuming high-sugar and high-fat foods has significantly risen. Research indicates that high-sugar diets can lead to insulin resistance and fatty liver, thereby increasing the risk of DM (38). Moreover, high-fat diets not only cause weight gain but also affect insulin secretion and function (39). For instance, a study involving American adolescents found that those who consumed sugar-sweetened beverages daily had a significantly higher risk of obesity and T2DM compared to their peers who did not consume such beverages (40). Similarly, diets high in saturated and trans fats are associated with decreased insulin sensitivity and poor glycemic control (41). A prospective cohort study of adolescents also pointed out that a high-energy fast food diet is significantly associated with elevated fasting blood glucose levels and the occurrence of prediabetic symptoms (42).

Over the past few decades, the number of obese adolescents has been steadily increasing in many countries (43). Estimates for childhood obesity from 2017 to March 2020 show prevalence rates of 20.7% for children aged 6 to 11 and 22.2% for children aged 12 to 19 (44). As a major risk factor for the development of T2DM, the rise in body mass index (BMI) in adolescents is associated with an increased risk of being diagnosed with T2DM at a young age (45). Meta-analysis results for 228,184 participants show that the prevalence of T2DM in obese individuals is 1.3%, which is 13 times higher than in normal-weight individuals, and the prevalence of prediabetes is three times higher in obese individuals compared to normal individuals (46). Furthermore, a multicenter cross-sectional study on 2,448 adolescents with T2DM showed prevalence rates of 10.4% for overweight and 79.4% for obesity (47). Adolescents are in a transitional phase during puberty, making many patients simultaneously experience emotional disturbances. Current research indicates that emotional disorders can lead to the onset of diabetes and exacerbate its severity. The estimated prevalence of depression in T2DM patients is 25%, and it is associated with poorer blood glucose control in these patients (9, 48). An analysis of data from the Taiwan National Health Insurance Research Database from 2001 to 2010 found that, compared to reactive depression patients responsive to antidepressants, patients with treatment-resistant depression were more likely to be diagnosed with T2DM in later life (HR 1.51) (49). Additionally, obesity, IR, and hypoglycemic abnormalities may result in relatively poor cognitive function in adolescents with both T2DM and obesity (50).

## 3 IR affects the occurrence and development of YODM

### 3.1 IR

In addition to the continuous decline in function and quantity of pancreatic β-cells, tissue-specific IR is also a typical characteristic of T2DM. When the main target tissues of insulin, such as muscles, fat, and the liver, fail to respond appropriately to insulin, insulin sensitivity decreases continuously, making glucose less likely to be cleared from the bloodstream. Tissues begin to exhibit IR, leading to increased insulin production by the pancreas or presenting as hyperinsulinemia (51). Research has confirmed that IR plays a crucial role in the occurrence and development of various metabolic disorders, including non-alcoholic fatty liver disease (NAFLD), metabolic syndrome, obesity, hypertension, and cardiovascular diseases (52, 53). Chronic hyperinsulinemia resulting from IR can also lead to a reduction in pancreatic β-cell mass (54).

Currently, the primary mechanisms of IR involve abnormalities in substance metabolism, chronic inflammation, oxidative stress, among other aspects (55). Firstly, excessive energy intake promotes an increase in free fatty acids (FFA) and lipid deposition, leading to IR (56). AMP-activated protein kinase (AMPK), as an AMP-dependent protein kinase, is a key molecule in regulating biological energy metabolism. Studies have shown that activated AMPK can enhance insulin sensitivity and promote glucose uptake (57). Protein tyrosine phosphatase 1B (PTP1B), a widely expressed prototype non-receptor tyrosine phosphatase, is a critical negative regulator in insulin signal transduction. Overexpression in adipose tissue can lead to dephosphorylation of insulin receptors and inhibit insulin signal transduction (58). GLUT4 is an insulin-dependent transmembrane carrier protein that facilitates the translocation of glucose on the cell membrane. IR leads to reduced glucose uptake and is associated with impaired GLUT4 translocation (59).

Certainly, there is ample evidence indicating that chronic inflammation is pervasive throughout the course of DM, and it also influences IR (60). Macrophages can be classified into two different subtypes based on their activation status: M1 polarization, which promotes inflammation and is associated with reduced insulin sensitivity (61), and M2 polarization, which inhibits inflammation and is associated with enhanced insulin sensitivity (62). Activation of the NF-κB and JNK pathways due to pro-inflammatory status increases the secretion of various inflammatory mediators, including tumor necrosis factor-alpha (TNF-α), interleukin-1 beta (IL-1β), and interleukin-6 (IL-6), leading to the promotion of IR (63–65). Oxidative stress and inflammation often coexist and mutually influence each other in the host. Reactive oxygen species can stimulate the production of inflammatory factors, and, in turn, cellular inflammatory factors can promote the generation of oxygen free radicals (66). Oxidative stress reduces the translocation of IRS-1, decreases protein kinase B (PKB) phosphorylation, and lowers GLUT-4 expression (67, 68).

### 3.2 Key role of IR in the occurrence and development of YODM

**Figure 1:**
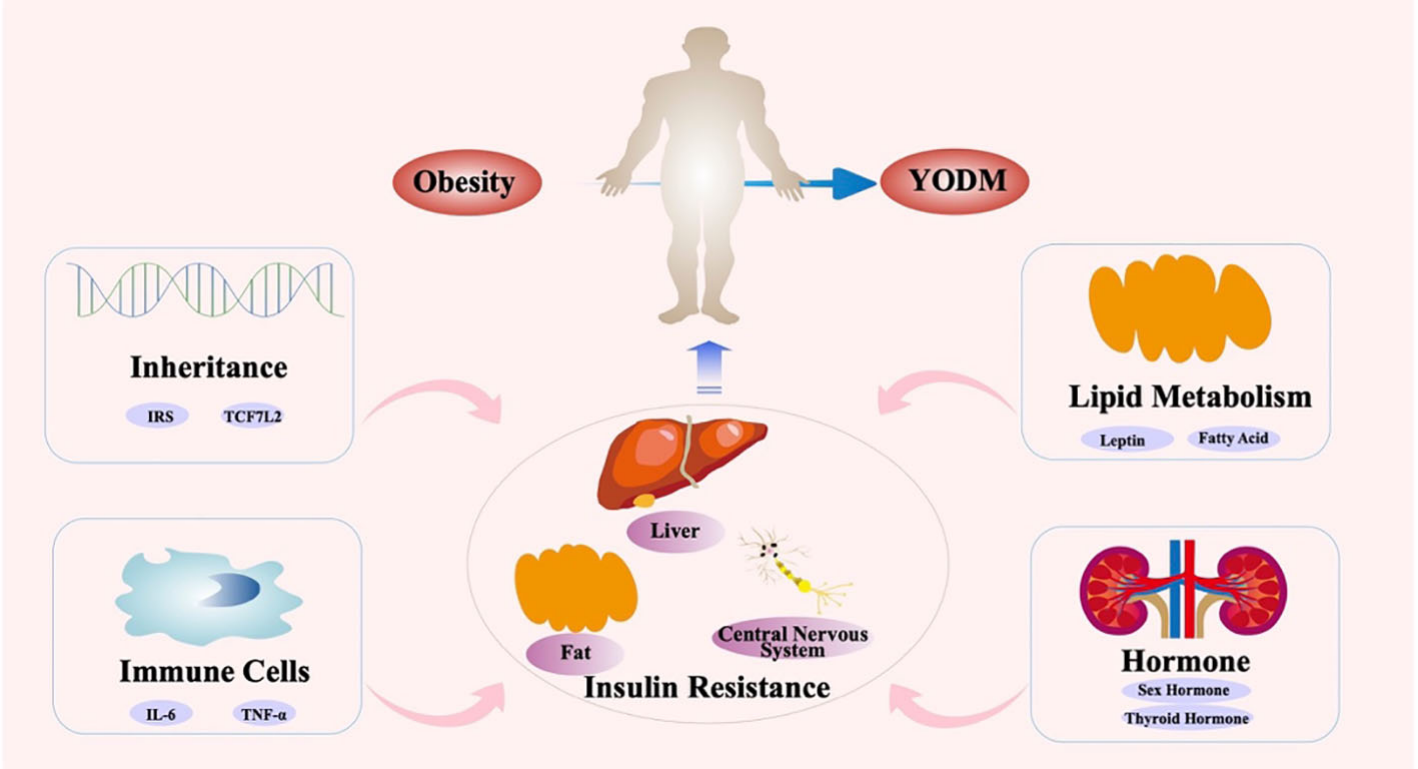
Key role of IR in the occurrence and development of YODM.

Existing clinical studies confirm that both insulin sensitivity and insulin secretion are impaired in YODM patients (69). Genetic-related β-cell dysfunction has been found in East Asian adolescents, and it is significantly associated with the development of T2DM even with mild decreases in insulin sensitivity (70). In contrast, IR is prevalent in the Indian population and may manifest as early as in neonates (71). The Restoring Insulin Secretion (RISE) study indicates that compared to adults with T2DM, YODM exhibits more severe IR, higher insulin secretion demand, and lower insulin clearance rates (15). A comparative study of YODM diagnosed within 1.5 years, matched with non-T2DM individuals in terms of BMI, showed a 75% decrease in the first-phase and a 55% decrease in the second-phase insulin secretion in YODM, accompanied by severe peripheral and hepatic IR (69). This may be attributed to hormonal changes affecting insulin sensitivity during puberty, and the more severe obesity issues in adolescent patients also affect peripheral tissue insulin sensitivity (72). IR may prompt β-cells to secrete more insulin, leading to earlier β-cell failure. Dysfunction, rather than death, is a common β-cell defect in T2D (73). Therefore, focusing on IR in the early stages of YODM and conducting intervention studies may improve β-cell function to some extent. A cross-sectional study, including 79 adolescents aged 10 to 18, assessed body composition indicators: body mass index (BMI), body fat percentage, waist circumference, and subcutaneous fat. IR was diagnosed in 29.1% of patients, and these body composition indicators were all correlated with IR (74) (**Figure 1**).

Considering the causes of obesity-induced DM, IR plays a crucial role (75, 76). A study conducted in Japan using intravenous glucose tolerance testing revealed IR in both obese non-diabetic adolescents and YODM individuals (77). Additionally, one of the major complications of obesity is IR, leading to disturbances in carbohydrate metabolism. Compared to normal-weight children, overweight and obese children have a higher incidence of IR (78). In the early stages of the disease, pancreatic β-cells can compensate for IR by increasing insulin secretion in the pathogenesis of glucose intolerance. Compensatory hyperinsulinemia induces increased appetite and weight gain. With the decline in pancreatic β-cell function and inadequate insulin secretion, the transition from IR to impaired glucose tolerance and then to T2DM occurs (79). Androutsos and colleagues applied principal component analysis to a health growth study cohort of over 2000 Greek children aged 9–13. They observed a positive correlation between a combination of high screen time, short sleep duration, and high sugary beverage consumption with HOMA-IR, while a combination of high to moderate-intensity physical activity and frequent eating showed a negative correlation with HOMA-IR (80). Dietary imbalance is prevalent in YODM and affects obesity and psychosocial function.

In the TODAY study, 20% and 6% of young people exhibited subclinical and clinical binge eating (5). In the SEARCH study, 50.3% of youth and YODM had dietary imbalances (81). Changes in gastric redoxin secretion may be an early biomarker of impaired glucose regulation in obese children with IR (82). Relevant studies found that for each additional hour of sedentary time and screen use between ages 8–10 and 15–17, insulin sensitivity decreased by 8.2% and 6.4%, respectively, while fasting blood glucose increased by 0.03 mmol/L and 0.02 mmol/L. This emphasized the evidence of sedentary behavior and screen time as key driving factors for the high-risk development of YODM (83).

Simultaneously, numerous factors, including genetics, inflammation and immunity, lipid metabolism, and adolescent hormones, affect signaling pathways such as insulin receptors, mediating the occurrence of IR. Firstly, certain genetic variations may lead to IR during adolescence. Studies indicate that polymorphisms in specific genes may influence key molecules in insulin signal transduction pathways, resulting in the occurrence of IR. The development of IR in YODM usually involves complex regulation by various genetic mechanisms. Genes of the insulin receptor substrate (IRS) family are closely associated with IR. Proteins encoded by IRS genes are critical molecules in insulin signal transduction pathways, and their abnormal function may lead to IR. Polymorphisms in IRS genes have been found to be associated with the development of IR in YODM.

Polymorphisms in inflammatory factor genes may be associated with IR in YODM. Chronic inflammation associated with obesity can lead to IR and β-cell dysfunction (84). Abnormal expression of inflammatory factors may increase tissue inflammatory responses, thereby interfering with the normal function of insulin signal pathways, leading to IR. During adolescence, changes in hormone levels and cytokines may increase adipose tissue inflammatory responses, further exacerbating the degree of IR. Inflammatory responses lead to increased release of cytokines such as tumor necrosis factor-alpha (TNF-α) and interleukin-6 (IL-6). These cytokines may directly interfere with insulin signal transduction pathways, affecting the function of insulin receptors and the effectiveness of insulin. The pro-inflammatory cytokine TNF-α can induce IR by disrupting early insulin-stimulated tyrosine phosphorylation (85). The increase in inflammatory reactions may induce autoimmune reactions, activate immune cells, disrupt immune regulation, and cause immune stress, leading to increased release of inflammatory factors, thus interfering with the normal function of insulin signal pathways. Activation of autoimmune reactions may damage and destroy insulin-secreting cells. Activation of immune cells such as macrophages and lymphocytes may increase the release of inflammatory factors, affecting the normal function of insulin signal pathways. Imbalance in immune regulation may lead to an increase in inflammatory reactions and abnormal activation of immune cells. Chronic immune stress may lead to increased activation of immune cells and increased release of inflammatory factors. All these abnormal changes can affect the normal function of insulin signal pathways.

Patients with YODM often exhibit lipid metabolism disorders (86, 87), including hyperinsulinemia and lipid peroxidation. These factors lead to the release of more fatty acids from adipocytes, further worsening IR and insufficient insulin secretion. IR can also lead to abnormalities in lipid metabolism, where fatty acids are not efficiently utilized by muscle and adipose tissues, leading to the synthesis of triglycerides in the liver, resulting in a vicious cycle of fatty liver and IR. The main cause of lipid metabolism disorders may be attributed to poor lifestyle habits during adolescence. A high-sugar, high-fat, high-energy-density diet may lead to weight gain and fat accumulation. Additionally, poor dietary structure may lead to abnormal expression of key molecules in the insulin signal pathway, thereby disrupting normal insulin signal transduction. Lack of physical exercise leads to reduced energy consumption in muscles, lipid metabolism disorders, and decreased insulin sensitivity. Moderate physical exercise helps control weight, improve the function of insulin signal pathways, and thus alleviate the degree of IR. Mechanistically, adipose tissue secretes various factors, including leptin, adiponectin, etc., which may negatively impact insulin sensitivity. The IR in YODM is related to the total body fat mass, especially visceral fat. This is mediated by various fat factors, including leptin, resistin, interleukin-6 (IL-6), and tumor necrosis factor-alpha (TNF-α). Additionally, non-esterified fatty acids contribute to IR by increasing hepatic glucose output and hindering glucose uptake in skeletal muscles (88, 89). Furthermore, an increase in visceral fat leads to decreased levels of adiponectin and reduced expression of adiponectin receptors on cell surfaces (90).

Adolescence is a critical period for growth and development, and changes in hormone levels during puberty have a significant impact on insulin sensitivity. During adolescence, changes in sex hormones may affect fat distribution and the growth and development of muscle tissue, influencing the function of insulin signal transduction pathways. Alternatively, hormonal changes during adolescence may affect IR through neuroendocrine regulatory mechanisms. Other factors, including changes in thyroid hormone levels and adrenal cortex hormone levels during adolescence, may also affect metabolic pathways and insulin signal transduction, thereby influencing the occurrence of IR.

## 4 Comprehensive management strategies for YODM from the perspective of IR

**Figure 2:**
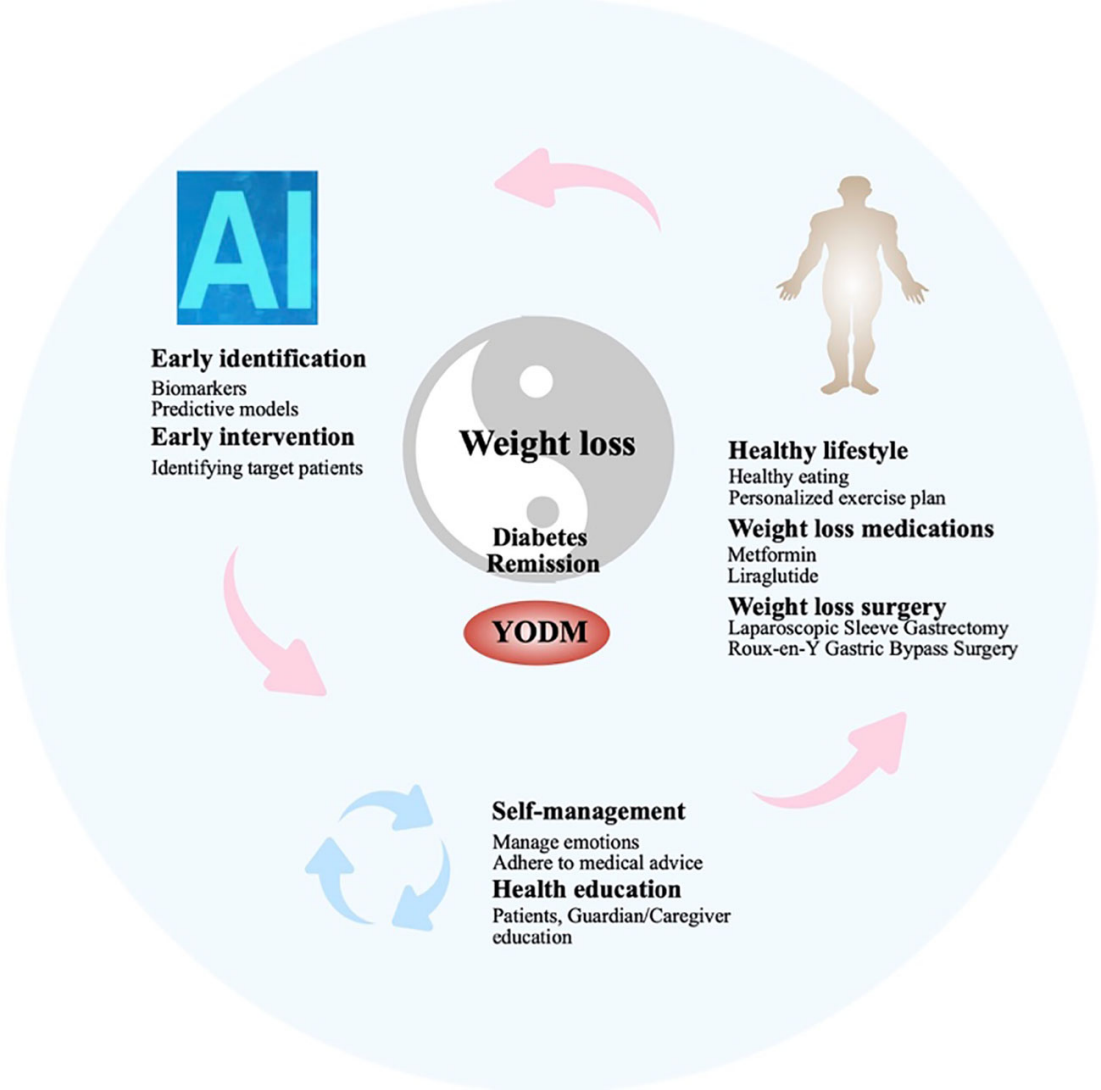
Comprehensive management strategies for YODM from the perspective of IR.

In 2016, the WHO’s “Global Diabetes Report” clearly stated that weight loss and restricted energy intake could lead to the remission of T2DM. In 2021, the ADA defined T2DM, recommending discontinuation of antidiabetic medications for at least 3 months, with a glycated hemoglobin (HbA1c) < 6.5% as the standard for T2DM remission. However, in certain situations, such as the presence of hemoglobin variants, diseases affecting red blood cell lifespan, or inaccuracies in HbA1c testing methods, HbA1c may not reflect true blood glucose levels. In such cases, FBG < 7.0 mmol/L or estimated HbA1c < 6.5% through continuous glucose monitoring (CGM) can be used as alternative criteria. There is clear clinical evidence supporting the significant reduction in weight and promotion of T2DM remission through enhanced lifestyle interventions or metabolic surgery in T2DM patients with a short course and obesity (91, 92). Additionally, short-term insulin therapy in newly diagnosed T2DM patients has also been shown to promote T2DM remission (93). While this evidence is focused on adults, it suggests that early intervention, including weight loss or medication targeting obesity, may be a crucial strategy for YODM to achieve diabetes remission (94). Therefore, early intervention strategies targeting IR, as a key factor in the occurrence and development of YODM, are crucial for achieving diabetes remission, with a focus on weight loss (95). Chronic hyperglycemia, or T2DM itself, is a significant cause of macrovascular and microvascular complications, including cardiovascular events, renal failure, and diabetes-related established microvascular and macrovascular damage leading to vision loss (96, 97). The remission of T2DM is associated with a lower risk of microvascular complications, especially in younger populations (under 45 years old) and those with fewer complications (none or less than 3) (98). Diabetes remission significantly helps improve patients’ quality of life and reduce the risk of complications. Therefore, the management strategy for YODM should shift some focus from controlling blood glucose and preventing complications to relieving diabetes. This should be the ideal treatment goal for such patients: early identification, early disease detection, precise screening of patients with well-functioning insulin beta cells, IR as the main pathological change, and obesity. This group is most likely to achieve diabetes remission or reversal through measures such as lifestyle changes, medication, or even surgery. Furthermore, considering that this population may have more emotional disorders or hormonal influences during adolescence, it is essential to provide health education to patients and caregivers, making them aware of the long-term benefits of maintaining good blood sugar control. In summary, the ideal goal is to adopt comprehensive measures to achieve diabetes remission or reversal, followed by controlling high blood sugar, obesity, and other risk factors associated with diabetes complications to delay the occurrence and progression of complications (**Figure 2**).

### 4.1 YODM patients experience faster disease progression, emphasizing the importance of early identification and early intervention

Early diagnosis and intervention help control the progression of the disease. In the early stages of diabetes diagnosis, effective treatment measures can improve the patient’s condition and contribute to diabetes remission. Approximately 40% of YODM cases are asymptomatic at the time of diagnosis, as diabetes is detected through screening due to signs of obesity, other risk factors, or IR (99). Therefore, early identification is crucial and can be achieved through alternative indicators such as waist circumference, triglyceride-glucose (TyG) index, metabolites, and IR index.

A study analyzing data from 38,000 Brazilian adolescents aged 12 to 17 compared the associations between obesity, overweight, waist circumference, and IR. The results showed that waist circumference is more helpful in identifying adolescents with IR, especially in late adolescence (100). Another multicenter cross-sectional study analyzed 161 newly diagnosed T2DM children and adolescents, with 1,935 children with normal blood sugar as healthy controls. The results indicated that the increase in HOMA2-B and HOMA2-IR is associated with a higher risk of T2DM, suggesting that besides IR, impaired beta-cell function is closely related to Chinese YODM. Gender differences in susceptibility and higher complications require YODM screening and prevention strategies (101). The triglyceride-glucose (TyG) index [area under the curve (AUC) 0.839)] showed better performance in identifying T2DM patients than HOMA-IR (AUC 0.645), demonstrating a significant association between IR and T2DM (102). Respiratory metabolites, including limonene, nonane, and 2,7-dimethylundecane, are correlated with IR evaluated by the homeostasis model assessment (HOMA-IR). Given their simple and non-invasive breath-based testing, these substances have the potential to serve as effective measures for early detection of insulin resistance (103). Another study tracking 190 newly diagnosed T2DM patients for 5 years identified 12 different metabolites, with forty-two hexaenoic acid, oxygen tri-nucleotide, dihydrocholesterol, and trienoic acid being the most representative (104).

In recent years, the global prevalence of T2DM has been increasing annually, with a significant contributing factor being the rising rates of overweight or obesity due to lifestyle changes. Clinical research results indicate that whether through lifestyle, medication, or metabolic surgical interventions, it is possible to slow down the progression from prediabetes to diabetes or achieve the reversal of elevated blood sugar, leading to normal levels, as T2DM remission (105, 106). Current research indicates that only some patients can achieve diabetes remission. These conditions include the exclusion of specific types of diabetes, such as secondary diabetes or diabetes caused by certain genetic factors, as well as patients with a longer course, more severe complications, and poor pancreatic beta-cell function. For T2DM patients without autoimmune destruction of pancreatic islets, overweight or obesity (BMI ≥ 25 kg/m^2^), preserved beta-cell function, and a course of ≤ 5 years have a higher likelihood of remission (91, 107). However, among these conditions, early-stage obesity serves as a crucial signal for achieving diabetes remission.

The Japan Diabetes Data Management (JDDM74) analyzed data from over 4,000 patients over 30 years nationwide, with 3,454 patients achieving diabetes remission, significantly correlated with a moderate weight loss of 3.0–7.9% (108). Therefore, based on the current clinical characteristics achievable for diabetic patients, combined with ongoing interventional research, weight loss and subsequent visceral fat loss are crucial for relieving T2DM, with obesity being the central core for achieving remission. Obesity can lead to excessive growth of adipose tissue, especially abdominal fat. This adipose tissue releases various hormones and cytokines, including free fatty acids, leptin, tumor necrosis factor, etc. These substances can interfere with insulin signaling, leading to insulin resistance. In the obese population, adipose tissue releases an increasing amount of non-esterified fatty acids, glycerol, hormones, pro-inflammatory cytokines, and other factors involved in the development of insulin resistance (109).

### 4.2 Poor adherence in YODM patients: cultivating effective self-management and improving adherence are key to diabetes management

Unlike adults, adolescents are more heavily influenced by external factors, making it challenging for many YODM patients to effectively control their diet, engage in physical activity, and manage blood glucose in their daily lives. Adolescents, being in a sensitive period of growth transition, often experience unstable behavioral and emotional states, hindering stable blood sugar control. However, the remission of diabetes may have more significant benefits for adolescents. Medical goals for YODM include preventing further weight gain and/or achieving weight loss (encouraging obese patients to lose 7–10% of their weight), maintaining good blood sugar control, reducing disease-related stress, and managing adolescent psychosocial issues (110).

For diabetes patients, especially adolescents, considerations should extend beyond the treatment of diabetes itself to include other conditions caused by diabetes, including the emotional disorders often associated with adolescents. Diabetes distress encompasses complications, self-management demands, unresponsive provider reactions, poor interpersonal relationships, and negative emotional responses to diabetes itself. Depression and diabetes distress screening scores are closely related to YODM, similar to adolescents with T1DM (111). YODM exposed to various stressful life events often experience reduced quality of life, depressive symptoms, and lower compliance with prescription oral medications (112). In the Pediatric Diabetes Consortium, 22% of YODM cases were found to have comorbid depression, with only 9% receiving treatment in the past 12 months (113). Compared to non-diabetic peers, YODM is more likely to experience emotional or anxiety disorders before and after diagnosis (114). Therefore, patients must possess good self-management skills, adhere to dietary controls, medication regimens, and regularly monitor blood glucose levels. Patients should comply with treatment plans, including regular medication intake, follow-up appointments, and actively collaborate with doctors in adjusting and improving treatment plans.

### 4.3 IR and obesity: reciprocal causation, choosing appropriate weight loss methods is a crucial means of improving IR and achieving diabetes remission

Controlling body weight, especially for obese diabetes patients, can improve insulin sensitivity, contributing to the remission of diabetes. Therefore, weight loss and subsequent reduction in visceral fat are key to diabetes remission (115). Compared to adolescents, pre-adolescent YODM patients face a higher risk of related incidence, emphasizing the importance of intervention measures to prevent and treat obesity in early childhood (116). Patients need to adhere to a healthy lifestyle, including regular physical exercise, maintaining healthy dietary habits, and avoiding unhealthy habits such as smoking and excessive alcohol consumption. The Population-based Panasonic cohort study in Japan, analyzing the relationship between changes and diabetes remission in 1,903 patients over a 5-year period, showed that weight loss of ≥3.9 kg or ≥5.0% in patients with BMI ≥ 25 kg/m^2^ (obesity) may be effective in relieving new-onset type 2 diabetes, excluding patients with BMI < 25 kg/m^2^ (117). Lifestyle changes are an important component of YODM weight management. The T2DM youth weight management guidelines recommend that adolescents reach at least a 7–10% weight reduction from their final adult height, with weight indexes below the 85th percentile for those still growing (118) (**Table 1**).

**Table 1:** Potential beneficial management strategies for YODM.

Management Strategies	Contents
Dietary Management	
Intermittent Fasting	Alternate-Day Fasting, 5:2 Diet, 16:8 Diet, Random Fasting
Low-Energy Diet	Daily intake of 800–1500 kcal; prioritize low-energy density foods; reduce intake of high-energy density foods; avoid sugary beverages; ensure adequate intake of protein, fiber, vitamins, and minerals to prevent malnutrition.
Low-carbohydrate Diet	Daily carbohydrate intake is typically kept between 20–100 grams, with specific amounts varying from person to person; increase intake of protein and healthy fats; choose low-carbohydrate vegetables; avoid high-sugar fruits, grains, starchy vegetables, and refined carbohydrates.
Mediterranean Diet	The main foods include plenty of fruits, vegetables, whole grains, legumes, nuts, and seeds; healthy fats primarily sourced from olive oil, nuts, and seeds; protein mainly from fish and seafood, moderate amounts of poultry, eggs, and dairy, with red meat and processed meats limited; moderate intake of low-fat dairy products; moderate consumption of red wine (optional), with water as the primary beverage; using herbs and spices instead of salt for seasoning; encouraging communal dining with family and friends to enjoy the mealtime experience.
** Exercise Methods**	Engage in moderate to vigorous physical activity for 30 to 60 minutes on 3 days per week.
Medication Therapy	
Insulin	Glargine; Getemir; Degludec
Biguanides	Metformin
GLP-1RA	Liraglutide; Dulaglutide; Exenatide
Sulfonylureas	Glimepiride; Glipizide; Glyburide
SGLT-2i	Dapagliflozin
DPP-4 Inhibitors	Sitagliptin, Linagliptin
**Weight Loss Surgery**	Laparoscopic Sleeve Gastrectomy; Roux-en-Y Gastric Bypass

While there is currently evidence supporting the effectiveness of certain treatments in adolescents with type 2 diabetes, we must also be mindful of the potential side effects associated with these treatments (119). These include the possibility of hypoglycemic reactions, gastrointestinal discomfort, drug allergies, and others. Additionally, weight-loss surgery may lead to postoperative malnutrition, gastric emptying disorders, abnormal insulin secretion, among other issues (120, 121). Considering the psychological sensitivity of patients, it is crucial to continuously monitor their psychological status when implementing treatment plans or making recommendations. Timely intervention for any emerging social and psychological barriers is also necessary.

#### 4.3.1 Dietary therapy

Dietary control can achieve standard weight and correct metabolic disorders. Dietary fiber can improve postprandial blood sugar and long-term diabetes control, with grain dietary fiber enhancing insulin sensitivity. As part of intensified lifestyle therapy, every T2DM patient should undergo comprehensive nutritional education (122). Reducing the consumption of sugary beverages, increasing the consumption of whole-grain bread and cereals, fruits, and vegetables is recommended (123). A randomized controlled clinical study in China confirmed that intermittent fasting intervention for 3 months could achieve diabetes remission for at least a year (124). A systematic review evaluating lifestyle changes (including three types of diets—low-energy diet, low-carbohydrate diet, Mediterranean diet, and exercise including aerobic and resistance sports, walking, and maintaining habitual physical activity) demonstrated effective diabetes remission, including weight loss and improved quality of life (125).

#### 4.3.2 Exercise therapy

Exercise plays a crucial role in the treatment of YODM, aiding in weight reduction, increasing insulin sensitivity, and enhancing peripheral tissue glucose uptake. The choice of exercise type and amount should be individualized based on gender, age, physique, physical strength, exercise habits, and preferences. Clear evidence indicates that regular physical activity benefits children and adolescents, including developing motor skills, promoting healthy weight and body composition, supporting bone and muscle development, and positively influencing insulin sensitivity (126, 127). Engaging in vigorous physical activity for 16 weeks twice a week significantly increased insulin sensitivity in overweight adolescents, independent of changes in total fat mass and lean tissue mass (128). The American Diabetes Association (ADA) and the International Society for Pediatric and Adolescent Diabetes (ISPAD) recommend encouraging adolescents with Youth-Onset Diabetes Mellitus (YODM) to engage in at least 60 minutes of moderate to vigorous physical activity daily, including muscle and bone strength training at least 3 days per week (129, 130). Exercise increases the expression of GLUT4 receptors, thereby enhancing peripheral glucose absorption. The benefits of improved insulin sensitivity persist for 16 hours after exercise in both healthy subjects and patients with Type 2 Diabetes Mellitus (T2DM) (131). In a cross-sectional study involving 164 YODM adolescents engaging in vigorous physical activity, lower HbA1c levels and better cardiovascular parameters were observed (132).

#### 4.3.3 Pharmacological treatment

Despite significant progress in the treatment of adult T2DM over the past few decades, the efficacy in YODM has seen only slight changes. Besides insulin, metformin is the first-line medication approved by the U.S. Food and Drug Administration (FDA) for treating YODM (118, 133). Although emerging therapies have provided new perspectives for managing YODM, metformin remains a cost-effective and safe part of treatment plans for many YODM patients globally due to its beneficial metabolic effects (134). However, the reality reflects that in almost half of the cases, metformin has been found insufficient for treating YODM (135, 136), especially in adolescents with severe metabolic disturbances at the time of diagnosis (137). Given that postprandial insulin release is often blunted or impaired in T2DM patients, GLP-1 agonists improve blood glucose control by enhancing postprandial insulin release. Additionally, these drugs slow gastric emptying, prolong beta-cell lifespan by inhibiting apoptosis, increase peripheral glucose uptake in muscles, and reduce hepatic glucose output, thereby improving insulin sensitivity. An indirect mechanism for improving insulin sensitivity is weight loss. The weight-loss effects of these drugs are primarily attributed to delayed gastric emptying and direct effects on suppressing appetite in the hypothalamus (138, 139). Compared to liraglutide, semaglutide has shown promising weight-loss effects in adults with obesity and T2DM (140, 141). Until July 2019, the GLP-1 receptor agonist liraglutide was approved by the FDA for treating YODM and/or obesity in individuals aged 10 and above (142, 143). However, the approved dose for weight management (3 mg per day) is higher than the dose used for managing T2DM (1.8 mg per day). In a systematic review of 9 randomized controlled trials involving 286 obese, prediabetic, or YODM individuals aged 18 and under, GLP-1 agonist use was associated with an average weight loss of 2.7 kg. This effect was observed more in patients with higher BMI (144). A randomized controlled trial showed a favorable impact on YODM weight when GLP-1 agonists were used in conjunction with metformin. In a study of 134 children and adolescents (10–17 years) with T2DM, the liraglutide group experienced a weight loss of 2.3 kg at week 26, while the placebo group lost 0.99 kg (145). An investigation in Korea assessed the efficacy and side effects of once-weekly dulaglutide in treating YODM. The results indicated excellent glycemic control with once-weekly dulaglutide for YODM, with no significant side effects (146). However, other studies found additional side effects of GLP-1 agonists, including vomiting, diarrhea, abdominal pain, hypoglycemia, elevated transaminases, and pancreatitis (147). The use of sulfonylurea drugs in children effectively lowers HbA1c by 1.5–2% and has been approved in several countries. However, adverse reactions, including weight gain, accelerated β-cell loss, and hypoglycemia leading to seizures and negative cognitive consequences, have been observed (148). An evaluation of the efficacy and safety of several glucose-lowering medications provides additional options for YODM management. Compared to FDA-approved drugs, dapagliflozin, sitagliptin+metformin, and saxagliptin+metformin showed better efficacy, indicating the top ten treatment methods for YODM in individuals aged 10–17: saxagliptin+metformin, liraglutide+metformin, liraglutide, dapagliflozin, exenatide-2 mcg, sitagliptin+metformin, linagliptin-5 mg, linagliptin-1 mg, metformin, and exenatide-5/10 mcg (149).

Implementing short-term intensified insulin therapy in uncontrolled hyperglycemic patients in the early stages of the disease improves β-cell function, ensuring remission of T2DM for a considerable period (150, 151). Insulin therapy is typically associated with weight gain (152, 153). Remission after intensified insulin therapy has been shown to last for over 2 years, and the shorter the time interval between diagnosis and intensified insulin therapy, the greater the likelihood of remission (154). According to the International Society for Pediatric and Adolescent Diabetes (ISPAD) guidelines, if HbA1c levels do not reach the target of 6.5–7% after 4 months of metformin treatment for YODM, initiating basal insulin at a dose of 1.5 IU/kg/day is recommended. If adding basal insulin is insufficient, prandial insulin at a dose of 0.1 U/kg/meal may be necessary (148).

#### 4.3.4 Bariatric surgical treatment

For patients who find it challenging to achieve weight loss after lifestyle interventions, or for those who prefer a more direct approach, bariatric surgery can be considered. The American Diabetes Association (ADA) recommends considering bariatric surgery for YODM patients with a BMI greater than 35 kg/m^2^, as surgery has a more favorable impact on blood glucose control (including HBG, HbA1c, HOMA-IR) compared to medical treatments (155). Metabolic and bariatric surgery (MBS) has been associated with excellent weight loss and T2DM remission in adolescents (156, 157). The two most common surgeries for adolescents are laparoscopic sleeve gastrectomy (LSG) and Roux-en-Y gastric bypass. In the TEEN-LABS alliance study, among 242 adolescents undergoing MBS, the average weight decreased by 27% three years after surgical intervention. There was no significant difference in weight loss between patients undergoing Roux-en-Y gastric bypass (161 participants) or sleeve gastrectomy (67 participants) (28% and 26%, respectively). Furthermore, 95% (95% CI; 85 to 100) of patients with T2DM at the time of surgery achieved remission. Similarly, remission rates for other comorbidities were high (renal dysfunction at 86%, prediabetes at 76%, hypertension at 74%, dyslipidemia at 66%) (120). Laparoscopic sleeve gastrectomy (LSG) is gradually becoming a new benchmark for the treatment of morbid obesity and related complications in pediatric cases. A retrospective study involving 64 pediatric patients aged 5 to 14 provided further specific evidence of the beneficial metabolic effects of LSG surgery on morbidly obese children, effectively reducing complications related to Type 2 diabetes by lowering HbA1c levels after surgery (158).

## 5 Outlook and conclusion

Overall, based on the existing published research, there are still many areas that need improvement in screening, treatment, and other aspects of YODM. Given the continuously increasing number of YODM patients and the limited interventions available, there is a need to focus more on key aspects of the occurrence and development of YODM. This includes early identification and timely intervention, considering the unique characteristics of adolescents. For early identification, constructing predictive models that encompass various risk factors such as obesity, family history, unhealthy lifestyle habits, emotional disorders, along with data from metabolic and insulin resistance testing, may help identify patients early on. Specifically, in the future, leveraging new technologies such as artificial intelligence, big data, and others can provide more intelligent and precise support for early screening, diagnosis, and treatment of type 2 diabetes in adolescents. It is crucial not to wait until other chronic complications (such as peripheral neuropathy, kidney disease) or acute complications (such as ketoacidosis) emerge. Early intervention during this golden period is essential for achieving diabetes remission. In the future, precision medicine approaches such as genomics and metabolomics can be utilized to provide more personalized diagnosis and treatment for type 2 diabetes in adolescents, thereby improving treatment effectiveness and prognosis. Currently, the key interventions for diabetes patients consistently revolve around insulin resistance-mediated obesity, emphasizing the importance of maintaining normal weight. Good lifestyle habits are crucial in this regard and require supervision and monitoring from family and schools. Indeed, strengthening health education and intervention strategies across multiple levels such as family, school, and community can promote the adoption of healthy lifestyles among adolescents, thereby preventing and controlling the occurrence and progression of type 2 diabetes. Medications such as GLP-1 agonists can serve as effective supplementary interventions. For some patients, bariatric surgery may be more suitable and effective. Of course, in the future, precision medicine approaches such as genomics and metabolomics can be utilized to provide more personalized diagnosis and treatment for type 2 diabetes in adolescents, thereby improving treatment effectiveness and prognosis.

In summary, early identification, early intervention, and effective evaluation centered around IR in YODM can be crucial for achieving diabetes remission. However, healthcare professionals and researchers may significantly help individuals with YODM by identifying and implementing better strategies for improving insulin resistance and intervening in obesity at an early stage.

## Author contributions

QJ: Writing – original draft, Writing – review & editing. YZ: Writing – original draft. BZ: Conceptualization, Writing – review & editing. XA: Conceptualization, Writing – original draft, Writing – review & editing.
